# What is presumed when we presume consent?

**DOI:** 10.1186/1472-6939-9-8

**Published:** 2008-04-25

**Authors:** Barbara K Pierscionek

**Affiliations:** 1 Department of Biomedical Sciences, University of Ulster, Cromore Road, Coleraine, BT52 1SA, UK

## Abstract

**Background:**

The organ donor shortfall in the UK has prompted calls to introduce legislation to allow for presumed consent: if there is no explicit objection to donation of an organ, consent should be presumed. The current debate has not taken in account accepted meanings of presumption in law and science and the consequences for rights of ownership that would arise should presumed consent become law. In addition, arguments revolve around the rights of the competent autonomous adult but do not always consider the more serious implications for children or the disabled.

**Discussion:**

Any action or decision made on a presumption is accepted in law and science as one based on judgement of a provisional situation. It should therefore allow the possibility of reversing the action or decision. Presumed consent to organ donation will not permit such reversal. Placing prime importance on the functionality of body organs and their capacity to sustain life rather than on explicit consent of the individual will lead to further debate about rights of ownership and potentially to questions about financial incentives and to whom benefits should accrue. Factors that influence donor rates are not fully understood and attitudes of the public to presumed consent require further investigation. Presuming consent will also necessitate considering how such a measure would be applied in situations involving children and mentally incompetent adults.

**Summary:**

The presumption of consent to organ donation cannot be understood in the same way as is presumption when applied to science or law. Consideration should be given to the consequences of presuming consent and to the questions of ownership and organ monetary value as these questions are likely to arise should presumed consent be permitted. In addition, the implications of presumed consent on children and adults who are unable to object to organ donation, requires serious contemplation if these most vulnerable members of society are to be protected.

## Background

The controversy about presumed consent has been recently revived in the UK as a consequence of the organ donor shortfall [1,2]. The debate is far from settled and only altering the law to permit the presumption of consent with respect to donor organs will provide the answer to whether this will indeed remedy the problem of organ shortage. It will not, however, answer the questions about what exactly is being presumed; the ethical and legal implications that could follow will need to be considered carefully. The meaning of presumption, the issues that could be raised about the ownership of and rights over body organs and further implications for gain, are discussed. Applications of presumed consent in different countries are compared and the need for further investigation of factors that influence donation and of attitudes towards presumed consent, is acknowledged. This article also addresses the issue of protection of the most vulnerable members of society who may be unable to dissent.

## Discussion

### The meaning of 'presumed consent'

The understanding of presumption of consent to organ donation may be considered, by some practitioners of law or science, to be an inaccurate and misleading term. This stems from the general understanding of 'presumption' in law and science: as an inference that is made on available fact or evidence with the understanding that vital information that can render the inference invalid may be missing. In law a presumption holds – that of innocence, for example – until a substantial body of evidence is produced to the contrary. Just like a scientific theory or hypothesis, a legal presumption is maintained for as long as no evidence is provided to disprove it or no valid objection is raised against it. A presumption, in law and science, is therefore a 'provisional estimate of facts' [3] based on some accepted fundamental state or pattern of behaviour.

Unlike the presumptions in law or the hypotheses of science, presumption of consent for the use of body organs cannot afford any possibility of abandoning the presumption, reversing the decision or of retracting any action based on the decision (clearly, the deceased donor cannot raise objections). The presumption of consent for organ donation cannot therefore be taken as a presumption of donor willingness, with the specific understanding that there will be a provision for changing the course of action should further evidence emerge, but rather as a presumption of state rights to post-mortem body organs, unless an objection by the 'occupant' of the body is raised whilst the 'occupant' is still in 'residence'. Opponents of presumed consent argue that the absence of donor willingness is morally unacceptable because it can be seen as a violation of their wishes [4]. (It has even been suggested that the term presumed consent be replaced by 'specified refusal' to put the emphasis on the action taken rather than on assumption [5]).

It is clear that presumed consent is advocated as a means of meeting organ donor shortages and not because the state wishes to assume ownership of body parts *per se*. Nevertheless, it places the greater emphasis on functionality of body organs and how they can be best utilised to sustain life rather than on the importance of requiring permission of the individual to donate his or her organs. It also takes away the power to 'gift' that donorship confers [6].

If functionality of body organs becomes of prevailing importance, it could be argued that the body is predominantly a vessel equipped with all the necessary instrumentation for maintaining life and that is occupied and used by the person to whom the body belongs. Consequently, if presumed consent is advocated, it could be reasoned that since after death the 'occupant' no longer needs the 'vessel', if any of the instrumentation is still functional it should be used to better or save the life of another. The acceptance of this premise and hence of the liberty of the state to assume the rights to decide about further usage (pending no objections) raises further issues about the right of ownership and hence who should benefit from body organs, and how presumed consent will extend to competent minors and mentally incompetent adults.

### The right to sell body parts

The debate about presumed consent and importance of optimising the functionality of body organs can extend to ownership and the right to sell these organs [7-9]. It has even been argued, in accordance with a rights-based theory of justice, that body organs are akin to goods to which a person can claim rights [10]; the prospect of treating body organs, in future, as 'property' has been raised in a common law case [11]. Whilst alive, the 'occupant' has an acknowledged right of ownership as evidenced in medical and other research practice: autonomy of mentally competent adults is respected and consent to medical treatment and/or study on any part of the body must always be sought. It could be argued that consent is required for research and medical treatment because there may be risks involved and that these risks no longer apply post-mortem, but this does not take into account all the underlying reasons for consent. One of these is the fundamental respect for the right of the individual to make decisions about his or her own body and in certain situations to accept payment for its usage as is evidenced when remuneration is offered for participation in research. It is not a quantum leap to suggest that if functionality of body organs is so valued and remuneration for research on the living body permitted, the worth of body organs (just like land and other acquired assets), should be allowed, after death, to pass to relatives or any other beneficiaries nominated by the deceased in a will. Indeed, this may be considered a natural extension of current practice: that next of kin take care of the deceased body [12]. If the state wishes to presume consent to organ donation (in the absence of any objections) and to treat these as transferable resources, the question of why individuals should not be permitted to organise for the sale of their body organs post-mortem, for the financial benefit of family and loved ones, could very well be raised. The sale of body organs is highly contentious but paradoxically, such a debate may raise awareness of the need for organs and the suggestion of a financial incentive could even encourage organ donation [7,8]. James Stacey Taylor [9] points out that any suggestion of establishing a market in body organs needs to be seen as a means of increasing supply for the benefit of those who are in need and therefore has sound moral grounding [9]. It should be considered alongside other measures and be properly controlled and implemented to avoid exploitation [9]. These arguments in support of the sale of body parts, are made with the assumption that what is considered to be 'morally permissible' does not take into account any objections based on religious beliefs [8]. Religious and cultural views need to be given just consideration because certain beliefs and traditions oppose the sale of body organs and indeed the notion of presumed consent [13]. Conversely, for others such measures are acceptable; surgeons from India have protested against the objections to body organ trade [14].

### Presumed consent in the case of children

The age at which autonomy is granted varies in the UK depending on whether it is with regard to consent to medical treatment or consent to participation in research. In the former case, children are given the status of autonomous adults at the age of sixteen; in the latter the age of consent is eighteen. This distinction has not been properly qualified in the UK and can lead to bizarre situations [15]. Before presumed consent can be permitted, there would need to be a decision made about the age at which autonomy of an individual to object to organ donation is respected. Once such an age is decided, it will inevitably lead, in the UK, to the question of Gillick competency. This term arose from English case law and is applied in relation to consent to medical treatment where there is a question about the rights of a minor to make autonomous decisions about such matters [16]. A Gillick competent child is one who, although chronologically below the determined age of consenting to medical treatment, is deemed to be intelligent and mature enough to understand the consequences of such a treatment and therefore is considered as able to be treated as an autonomous adult [16]. Whilst the notion of Gillick competency arose from the law, the decision about whether or not to treat a child as Gillick competent (and hence as an autonomous adult) is left to the medical practitioner. The decision about whether or not to allow a minor the autonomous status of an adult with regard to medical treatment is fraught with difficulty and uncertainties. It would become even more complicated if it were applied to presuming consent to organ donation, for the differences between appreciating the consequences of medical treatment, and hence being able to consent to it, and being sufficiently competent to understand what refusal of organ donation may mean, are vast. The former requires the young person to have an understanding of what the treatment will mean to him or her. The latter requires a much more evolved perspective on fundamental issues of life and death and the ability to make a balanced judgment and decision without being pressured by feelings of guilt that refusal will deprive another human being of life. It may be considered safer to reject the notion of Gillick competency for presumed consent altogether and leave the right of refusal for minors in all cases up to parents, guardians and carers. This does not simplify matters. Reliance solely on the decision of parents or guardians, could mean ignoring the wishes of a fourteen year old mother, who is estranged from her own parents and who on her death bed insists that she does not want any of her organs donated. (It is recognised that this situation can only occur where organ recovery is to be made without brain stem death). If Gillick competency to presumed consent cannot be invoked, it would permit taking organs from a minor in cases where no autonomous adult authorised to object to such a measure were available.

### Presumed consent in the case of mentally incompetent adults

Decisions on behalf of the mentally incompetent need to be made in cases of medical treatment and in such situations, common law in the UK sets the principle that such decisions should always be made in the best interests of the patient [17]. There have been instances in which it has been difficult to ascertain exactly what these best interests are. In one of the more controversial legal cases [18], the decision was made that it would be in best interests of Y, a physically and mentally handicapped woman, to transplant her bone marrow in order to save the life of her (mentally competent) sister even though the procedure was not without risk and provided no benefit to Y. The justification given for why this was in Y's best interests was that the sister had a young child who, without its mother, would be left in the care of its grandmother (Y's mother) who would then not be available to see Y as often as she did. Notwithstanding whether or not this explanation justified the best interests argument, it emphasizes the vulnerability of the mentally incompetent to the decisions that can be made whilst they are alive. The situation is even more uncertain after death. The 'best interests' argument that is used in the case of a living donor cannot be applied post-mortem. A mentally incompetent adult will be unable to raise objections to organ donation because, like a child, the mentally incompetent adult is deemed to be unable to understand the concept. However, unlike the child with parents who are able to make a decision about refusing donation, the mentally incompetent adult may have no living relatives able or permitted to make such a refusal on his/her behalf. The vulnerability of these individuals highlights one of the most potentially dangerous aspects of presumed consent. If presumption of consent is permitted, provision would need to be made to protect such individuals in order to avoid inadvertently exploiting the mentally incompetent as reservoirs of body organs.

### Applications of presumed consent

Introduction of presumed consent into law can take a number of forms, and variations in the application of presumed consent [19] have been broadly classed as 'strong' ('hard') or 'soft' [7,19] depending essentially on whether or not permission of relatives is required. However, the distinction between the options is blurred. Even in countries in which presumption of consent does not officially require the permission of relatives, this is sought and taken into account [11,19]. There is no conclusive evidence showing which option is the more successful in obtaining donor organs [2]. In France and Spain, where consent of relatives is routinely sought, the donor rates, in 2005, were 22.2 and 35.1 per million population, respectively; in Austria where consent of relatives is not routinely sought, the figure was 24.8 per million population [2]. It is worth noting that in Spain, organs are taken from heart-beating and non heart-beating donors and this has been credited with increasing donation rates [2]. With limitations, in the UK, on organ availability from donors who have suffered brain stem death (heart-beating donors), measures to increase the number of organs retrieved from non heart-beating donors are being advocated [20,21].

According to one mathematical model, organ availability is likely to be higher when presumed consent measures are introduced, even when other confounding factors are taken into account, but ambiguities in the model are acknowledged [19]. Results of a study on twenty-two countries show that whilst the highest rates of donation are found in countries like Spain, Austria, Latvia, Portugal and Belgium where presumed consent operates, there is a greater rate of donation (per million population) from Ireland (where informed consent is required) than from France, Slovenia, Czech Republic, Hungary and Italy where presumed consent applies [19]. The factors that influence donation have not been fully identified. It is notable that, with some exceptions, the Catholic countries of Europe, where the legal system is based on Roman law, presume consent [19,22,23] and stress the importance of 'for the greater good' [23]. In Protestant countries, individual rights are considered foremost and informed consent is more likely to be required [23].

A recent study [7] that investigated attitudes to presumed consent in Scotland found that, overall, a 'soft' option was favoured although, when it came to opinions from members of donor families, the views were not consistent. Other responses, suggest that a deeper probing of attitudes to presumed consent is needed. For example, while payment for organs was considered, by 70% of respondents, to be a measure that would encourage others to donate, only 21% agreed that a financial incentive should be used [7].

Ultimately, whether consent is express or presumed, the donor and/or relatives should be fully informed before any consent is obtained. Special attention needs to be given to those for whom others make decisions concerning their health and welfare. The easiest option would be to apply presumed consent only to specified groups (e.g. autonomous adults) and to disallow any vulnerable individuals from being considered as members of these groups. This would ensure that no situation of abuse of the vulnerable, whether intentional or unintentional, occurs. There is no certainty, however, that such a measure would not invoke cries of discrimination from those who may misinterpret the measure as an indication that the body organs of such individuals are of lesser value. It may also create difficulties and lead to complicated legal arguments with cases brought before the courts should a family situation arise that is similar to that of the case of Y [18], but with a deceased individual, who had been deemed unable to make their own decisions whilst alive, and whose organs could save the life of a relative.

The issues for vulnerable individuals require further analysis and, prior to the introduction of any legislation that allows for presumed consent, there needs to be a thorough re-examination of current laws that treat children and mentally incompetent adults as non-autonomous. With regard to children, reviews of the age of consent and the notion of Gillick competency with better guidelines for practitioners are needed. When deciding on mental impairment or incapacity, there should be an assessment of its scope, its nature, its duration and its durability (drugs, medications and alcohol can all have temporary effects that could render a person medically incompetent to make decisions but these effects may not be lasting).

## Conclusion

To presume consent may or may not alleviate the shortage of donor organs but it will most certainly raise a number of related ethical and legal complexities that will need to be addressed in order to safeguard against unacceptable practices. Fundamentally, what is meant by presumption and how this is applied to the concept of presumed consent to organ donation will have to be determined. It will need to extend beyond the boundaries of current legal and scientific notions and will involve legal and ethical arguments about the sale of body organs, rights of ownership, donation and bequeathing to beneficiaries. More investigation of attitudes towards presumed consent and why and how these may vary is required. Consideration must be given to objections on religious or cultural grounds and whether and how prospective legislation could impinge on beliefs and practices. As always, protection of the most vulnerable, unable to make autonomous decisions, is of paramount importance in any actions taken. Current laws and practices relating to consent and decision-making capacities of children and vulnerable adults need to be reviewed before introducing legislation that permits the presumption of consent.

## Summary

Consent for body organs cannot be presumed in the same way that presumption is used in law or science. Presuming consent for organ donation places the value of body organ function above the requirement for permission from the donor and raises a number of related ethical and legal questions about ownership and sale of body organs, rights of refusal for children and mentally incompetent adults. The factors that influence donation rates and attitudes to presumed consent have not been clearly identified and require further investigation. Measures for the protection of the most vulnerable need to be addressed before legislation to allow for presumed consent can be permitted.

## Competing interest statement

The author declares that she has no competing interests.

## Pre-publication history

The pre-publication history for this paper can be accessed here:


